# Supplementary material for: Reduced glucosinolate content in oilseed rape (Brassica napus L.) by random mutagenesis of BnMYB28 and BnCYP79F1 genes
Source: Sci Rep. 2023 Feb 9;13:2344. doi: 10.1038/s41598-023-28661-6 (PMC9911628; doi:10.1038/s41598-023-28661-6)
Supplement: Supplementary file 1 — Supplementary Tables. [file 41598_2023_28661_MOESM1_ESM.pdf]

Supplementary Tables

Supplementary Table S1: List of primers developed for RT-qPCR analyses.

Gene name	Primer name	Primer sequence (5‘ → 3‘)	Tm (°C)	Amplicon length (bp)
BnMYB28.C09	GSL_rt_2_6_F	acagttcacgcctcttactcc	60	144
	GSL_2_6_R	gcttcatgcttctcgtgga		
BnMYB28.A03	GSL_2_5_F	gcttcatgcttctcgtggt	64	194
	GSL_2_2_R	gcaaattctctggaggcgtgttgaca		
BnMYB28.Cnn	GSL_2_3_F	cgaacagggtattgatccca	58	264
	GSL_rt_2_3_R	gtgaccttagccgcaactttg		
BnCYP79F1.C05	GSL_3_1_F	cgccggaacacacgccatca	64	258
	GSL_rt_3_1_R	gcaaggaggttgtccgcttcaat		
BnCYP79F1.A06	GSL_rt_3_2_F	ggtatacaaaccagagcgtcacctc	64	222
	GSL_3_2_R	cctctagacttaacggtccg		
BnACTIN2	ACT1_F	tctggtgatggtgtgtctca	60	141
	ACT2_R	ggtcaacatgtaccctctctcg		
BnGAPDH	GC1_F	ccgcttgcttcaacatcatt	60	160
	GC2_R	tctgaggtctttcgacgctg		

Primers were designed based on the Darmor-*bzh* reference genome (Genoscope).
F= Forward primer, R= Reverse primer

Supplementary Table S2: List of primers used for generating amplicons for mutant screening. One amplicon per paralog was used for mutant detection.

		Standard PCR [1]		TILLING PCR [2]			
***B. napus* gene name**	**Darmor ID**	**Primer name**	**Primer sequence (5‘ → 3‘)**	**TILLING primer name**	**Primer sequence (5‘ → 3‘)**	**TILLING amplicon length (bp) [3]**	**cDNA coverage [4]**
BnMYB28.C09	*BnaC09g05300D*	SJ_0082	acagttcacgcctcttactcc	SJ_0094	gagcttctctattctcatcctag	902	65%
		SJ_0083	ggcttgtgagtcacgggatcag	SJ_0095	gaccgaccacctaaagaccag		
BnMYB28.A03	*BnaA03g40190D*	SJ_0084	catgaaaacaccttgcagcta	SJ_0096	gcattcttgggtgtttttgaggg	1,706	73%
		SJ_0085	ctcgggattactaaccctgaaggc	SJ_0097	gcgttggaactatcctcttc		
BnCYP79F1.C05	*BnaC05g12520D*	SJ_0086	gacaatgatgatgagccttacc	SJ_0098	ccgacttcgcagaccggcct	1,516	59%
		SJ_0087	cctctagacttaacggtcca	SJ_0099	cggcctattccaggacgg		
BnCYP79F1.A06	*BnaA06g11010D*	SJ_0088	gttgtttggaaggagacatatc	SJ_0100	cattgacgagagggtggagc	1,180	38%
		SJ_0089	cctctagacttaacggtccg	SJ_0101	ccatcatgatcgtcccgactttg		

[1] Standard PCR was done using unlabeled primers.

[2] TILLING PCR was done using a combination of unlabeled and infrared (IR) labeled primers. Forward and reverse primers were labeled with DY681 and DY781 probes, respectively. The TILLING PCR was done as a nested PCR using amplicons from the standard PCR as the template.

[3] Length of the amplicon analyzed for detection of EMS-induced mutations.

[4] Share of the total cDNA encompassed in the analyzed amplicon for detection of EMS-induced mutations.

Supplementary Table S3: HPLC analysis using commercial glucosinolate standards.

Compound	Side chain	Retention Time (min)	HPLC calibration (linear regression) [a]
Glucoiberin	3-me-sulfinylpropyl	5.79	(area-3367)/162966
Progoitrin	(*2R*)-2 hydroxy-3-butenyl	6.43	(area+1631)/164513
Epiprogoitrin	(*2S*)-2 hydroxy-3-butenyl	6.60	(area+18347)/157248
Sinigrin	2-propenyl	6.66	(area-11372)/199282
Glucoraphanin	4-me-sulfinylbutyl	6.65	(area+19466)/174317
Gluconapin	3-butenyl	7.69	(area-5783)/175044
Glucobrassicanapin	4-pentenyl	8.77	(area-9690)/162867
Glucotropaeolin	benzyl	8.91	(area-9122)/198955
Glucobrassicin	3-Indolylmethyl	9.40	(area-11784)/552644
Gluconasturtiin	phenethyl	10.00	(area-5888)/164863

[a] conversion from (area under the peak) to (nmol GSL)

Supplementary Table S4: All EMS-induced mutations detected in two *BnMYB28* and two *BnCYP79F1* paralogs. Mutation positions are relative to the translation start site.

B. napus **gene name**	**M_2 plant name** [1]	**M_2 mutant zygosity** [2]	**Mutation position on gDNA**	**cDNA change**	**Amino acid change**	**Mutation type**
BnMYB28.C09	70_F4	Hom	G 12 C	G 12 C	K 4 N	Missense
	70_H4	Het	G 12 C	G 12 C	K 4 N	Missense
	49_F2	Het	G 26 A	G 26 A	G 9 E	Missense
	66_B11	Het	G 28 A	G 28 A	E 10 K	Missense
	71_B4	Hom	G 28 A	G 28 A	E 10 K	Missense
	72_E9	Het	G 31 A	G 31 A	G 11 R	Missense
	71_E9	Het	G 43 A	G 43 A	G 15 R	Missense
	71_H9	Hom	G 43 A	G 43 A	G 15 R	Missense
	8_E5	Het	G 43 A	G 43 A	G 43 R	Missense
	64_C10	Het	G 45 A	G 45 A	A 15 A	Silent
	3_H2	Het	G 51 A	G 51 A	W 17 *	Nonsense
	11_H7	Het	G 51 A	G 51 A	W 17 *	Nonsense
	54_F10	Het	C 78 T	C 78 T	I 26 I	Silent
	43_E3	Hom	G 97 A	G 97 A	G 33 R	Missense
	54_C6	Het	G 98 A	G 98 A	G 33 E	Missense
	12_A9	Hom	C 122 T	C 122 T	P 41 L	Missense
	54_B9	Het	G 133 A	G 133 A	G 45 R	Missense
	54_C6	Het	G 250 A	G 149 A	G 50 E	Missense
	7_C6	Het	C 289 T	C 188 T	P 63 L	Missense
	7_D6	Hom	C 289 T	C 188 T	P 63 L	Missense
	76_D11	Hom	G 303 A	G 202 A	G 68 S	Missense
	64_A8	Het	G 303 A	G 202 A	G 68 S	Missense
	68_H6	Hom	G 303 A	G 202 A	G 68 S	Missense
	68_A11	Het	G 323 A	G 222 A	E 74 E	Silent
	45_B8	Het	C 335 T	C 234 T	I 78 I	Silent
	11_A5	Het	G 329 A	G 329 A	Q 228 Q	Silent
BnMYB28.A03	54_F3	Hom	G 50 A	G 50 A	W 17 *	Nonsense
	48_C8	Hom	G 51 A	G 51 A	W 17 *	Nonsense
	48_D8	Hom	G 51 A	G 51 A	W 17 *	Nonsense
	54_E1	Het	G 61 A	G 61 A	E 21 K	Missense
	48_H3	Het	G 223 A	G 134 A	G 45 E	Missense
	48_D3	Hom	G 317 A	G 228 A	Q 76 Q	Silent

	50_E3	Het	C 706 T	C 421 T	P 141 S	Missense
	56_E12	Hom	G 737 A	G 452 A	S 151 N	Missense
	50_E3	Het	C 899 T	C 614 T	T 205 I	Missense
BnCYP79F1.C05	52_C8	Het	C 424 T	C 424 T	E 142 *	Nonsense
	64_C7	Hom	C 560 T	C 560 T	T 187 I	Missense
	64_F6	Het	C 606 T	C 606 T	T 202 T	Silent
	54_A6	Het	C 654 T	C 654 T	F 218 F	Silent
	53_B5	Hom	G 770 A	G 770 A	G 257 D	Missense
	63_E7	Hom	G 850 A	G 850 A	E 284 K	Missense
	65_E3	Hom	C 956 T	C 956 T	P 319 L	Missense
	65_A8	Het	C 1399 T	C 1107 T	D 369 D	Silent
	66_F6	Het	G 1401 A	G 1109 A	R 370 K	Missense
	53_C5	Het	C 1430 T	C 1138 T	L 380 L	Silent
	53_D5	Het	C 1430 T	C 1138 T	L 380 L	Silent
	56_A4	Het	C 1430 T	C 1138 T	L 380 L	Silent
	61_A10	Het	C 1521 T	C 1229 T	T 410 I	Missense
	61_C10	Het	C 1521 T	C 1229 T	T 410 I	Missense
BnCYP79F1.A06	61_A2	Het	G 938 A	G 938 A	G 313 E	Missense
	45_D7	Het	G 1379 A	G 1090 A	E 364 K	Missense
	41_A5	Het	G 1384 A	G 1095 A	V 365 V	Silent
	46_B6	Het	G 1384 A	G 1095 A	V 365 V	Silent
	53_B9	Het	G 1385 A	G 1096 A	V 366 M	Missense
	53_C9	Het	G 1385 A	G 1096 A	V 366 M	Missense
	46_F5	Het	G 1394 A	G 1105 A	D 368 N	Missense
	44_F12	Het	G 1399 A	G 1110 A	R 370 R	Silent
	44_H12	Hom	G 1399 A	G 1110 A	R 370 R	Silent
	44_G12	Het	G 1399 A	G 1110 A	R 370 R	Silent
	63_G10	Het	C 1413 T	C 1124 T	S 375 F	Missense
	69_F7	Hom	C 1472 T	C 1183 T	P 395 S	Missense
	74_A7	Het	C 1472 T	C 1183 T	P 395 S	Missense
	69_H7	Het	C 1472 T	C 1183 T	P 395 S	Missense
	43_F5	Het	C 1473 T	C 1184 T	P 395 L	Missense
	43_G5	Het	C 1473 T	C 1184 T	P 395 L	Missense
	48_A12	Het	C 1473 T	C 1184 T	P 395 L	Missense
	48_D12	Het	C 1473 T	C 1184 T	P 395 L	Missense
	76_A4	Het	C 1473 T	C 1184 T	P 395 L	Missense
	75_B9	Het	C 1489 T	C 1200 T	V 400 V	Silent
	42_B3	Het	G 1511 A	G 1222 A	D 408 N	Missense

61_A2	Het	G 1511 A	G 1222 A	D 408 N	Missense
51_C7	Hom	C 1519 T	C 1230 T	T 410 T	Silent
55_B2	Hom	C 1520 T	C 1231 T	L 411 F	Missense
63_E8	Hom	C 1538 T	C 1249 T	P 417 S	Missense
44_G6	Het	G 1852 A	G 1289 A	G 430 D	Missense
44_H12	Hom	G 1898 A	G 1335 A	E 445 E	Silent
44_F12	Het	G 1898 A	G 1335 A	E 445 E	Silent
44_G12	Het	G 1898 A	G 1335 A	E 445 E	Silent

[1] Name of the single M_2 mutant as per the Express617 EMS mutant resource (Harloff et al., 2012).
[2] Zygosity of EMS-induced mutations observed in single M_2 individuals.
Silent mutations do not confer changes in polypeptide sequences.
* Premature stop codon mutation.

Supplementary Table S5: Summary statistics of functional effects conferred by the EMS-induced mutations in *BnMYB28* and *BnCYP79F1* paralogs. One amplicon per paralog was used for screening EMS-induced mutations.

			**Number of detected mutations**				
**Gene name**	**cDNA coverage**	**Number of M_2 pools screened [1]**	**Nonsense mutations**	**Missense mutations**	**Silent mutations**	**Splice site mutations**	**Mutation frequency (1/kb) [2]**
BnMYB28.C09	65%	13	2	19	5	0	1/37.8
BnMYB28.A03	73%	4	3	5	1	0	1/67.0
BnCYP79F1.C05	59%	4	1	7	6	0	1/38.0
BnCYP79F1.A06	38%	9	0	19	10	0	1/31.5
			Total = 78				Average = 1/43.5 kb

[1] The number of eight-fold (8x) two-dimensional (2-D) M_2 pools used for screenings.
[2] Calculated as the number of mutations per M_1 plant based on the number of analyzed M_2 families (8x 2D pools) and amplicon lengths.

Supplementary Table S6: Mutant genotyping in the M_3 generation to select crossing parents for the combination of single mutations.

								No. M_3 genotypes observed		
**M_3 seed code**	**Gene name**	**M_2 genotype**	**Germination rate (%)**	**Mutation position on gDNA [1]**	**cDNA change**	**AA change [2]**	**Number of M_3 plants genotyped**	**Homozygous WT**	**Heterozygous mutant**	**Homozygous mutants**
190623	*BnMYB28.C09*	$A_1A_eB_eB_e$	80	G 51 A	G 17 A	W 17 *	12	6	4	2
190624	*BnMYB28.C09*	$A_1A_eB_eB_e$	67	G 51 A	G 17 A	W 17 *	10	1	8	1
190625	*BnMYB28.A03*	$A_eA_eB_1B_1$	60	G 50 A	G 50 A	W 17 *	4	0	0	4
190628	*BnCYP79F1.C05*	$C_1C_eD_eD_e$	60	C 424 T	C 424 T	E 142 *	9	3	2	4
190630	*BnCYP79F1.A06*	$C_eC_eD_1D_1$	80	G 1379 A	G 1090 A	E 364 K	11	0	0	11

Plants were genotyped using Sanger sequencing of PCR fragments encompassing expected EMS-induced mutations.
All genotypes have been named as per designated allele codes (Table 2).
[1] Position of EMS induced mutation on the gDNA from the translational start site
[2] Position of amino acid change on the polypeptide chain
*Premature stop codon

Supplementary Table S7: Genotypes of parents used for hand crosses and the offspring derived thereof to combine single EMS mutants of *BnMYB28* and *BnCYP79F1* and genotypes of selected crossing parents and their progenies used for phenotyping experiments.

Crossing type	Parental genotypes	Genotype of selected F_1 progeny	F_2 seed code [a]	Genotypes used for phenotyping experiments [1]
M_3 x M_3	$A_eA_eB_1B_1$ x $A_1A_1B_eB_e$	$A_1A_eB_1B_e$	200527	$A_eA_eB_eB_e$, $A_1A_1B_eB_e$, $A_eA_eB_1B_1$, $A_1A_1B_1B_1$
	$C_eC_eD_1D_1$ x $C_1C_1D_eD_e$	$C_1C_eD_1D_e$	200529	$C_eC_eD_eD_e$, $C_1C_1D_eD_e$, $C_eC_eD_1D_1$, $C_1C_1D_1D_1$

All genotypes have been named as per designated allele codes (refer to Table 2).
Non-mutated wildtype alleles from Express617 are represented by the ‘e’ suffix in subscript.
[1] For quantitative and qualitative glucosinolate determination.
[a] Segregating F_2 population originating from heterozygous *BnMYB28* and *BnCYP79F1* double mutants (originating from direct M_3xM_3 crosses).

Supplementary Table S8: Summary statistics of individual glucosinolates identified with corresponding concentrations estimated in seeds of *BnMYB28* EMS mutants and controls. Analyses were done using high-performance liquid chromatography.

	**F_2 population [1]**					
	Population 200527				Control	
**Glucosinolate type [a]**	*BnMYB28*_DM	SD	*BnMYB28*_WT	SD	Express617	SD
Glucoiberin	0.53	0.25	0.30	0.08	0.10	0.01
Progoitrin	16.20	6.52	41.22	13.05	36.32	5.09
Epiprogoitrin	0.57	0.16	1.37	0.41	1.18	0.27
Sinigrin + Glucoraphanin	1.12	0.86	0.27	0.21	0.64	0.08
Gluconapoleiferin	0.04	0.02	0.11	0.07	n.d	
Glucoallysin	1.03	0.41	2.82	0.72	2.94	0.05
Gluconapin	6.05	2.70	5.66	1.06	9.93	2.52
4-Hydroxyglucobrassicin	2.56	0.61	2.31	0.16	2.23	0.32
Glucobrassicanapin	0.64	0.48	4.33	1.30	5.27	0.88
Glucotropaeolin	0.75	0.14	0.76	0.06	0.50	0.08
Glucobrassicin	0.26	0.12	0.13	0.03	0.20	0.36
Gluconasturtiin	1.08	0.24	1.07	0.13	0.73	0.16
**Sum (µmol/g DW)**	**30.83**		**60.35**		**60.04**	

[1] Experiments with plants originating from direct M_3xM_3 crosses without backcrossing.
[a] Estimated based on comparison of retention time and co-chromatography with commercial standards. GSL concentrations were calculated as µmol/g dry weight of tissue analyzed (five biological replicates per genotype).
*BnMYB28*_DM: *BnMYB28* double mutant (genotype $A_1A_1B_1B_1$), *BnMYB28*_WT: Plants with wildtype alleles for *BnMYB28* segregating within the same F_2 population, Express617: non-mutagenized parent
n.d: Not detectable SD: Standard deviation

Supplementary Table S9: Summary statistics of individual glucosinolates identified with corresponding concentrations estimated in seeds of *BnCYP79F1* EMS mutants and controls. Analyses were done using high-performance liquid chromatography.

	F_2 population [1]					
	Population 200529				Controls	
**Glucosinolate type [a]**	*BnCYP79F1*_DM	SD	*BnCYP79F1*_WT	SD	Express617	SD
Glucoiberin	0.01	0.01	0.04	0.02	0.10	0.01
Progoitrin	24.55	1.09	37.53	8.41	36.32	5.09
Epiprogoitrin	0.78	0.07	1.12	0.17	1.18	0.27
Sinigrin + Glucoraphanin	0.45	0.07	0.42	0.03	0.64	0.08
Gluconapoleiferin	0.08	0.02	0.05	0.02	n.d	
Glucoallysin	3.28	0.15	2.47	0.55	2.94	0.05
Gluconapin	6.91	0.20	8.91	0.73	9.93	2.52
4-Hydroxyglucobrassicin	4.40	0.19	3.81	0.68	2.23	0.32
Glucobrassicanapin	4.02	0.15	3.33	0.68	5.27	0.88
Glucotropaeolin	1.02	0.04	0.90	0.13	0.50	0.08
Glucobrassicin	0.31	0.05	0.19	0.06	0.20	0.36
Gluconasturtiin	1.62	0.07	1.56	0.13	0.73	0.16
**Sum (µmol/g DW)**	**47.43**		**60.33**		**60.04**	

[1] Experiments with plants originating from direct M_3xM_3 crosses without backcrossing.
[a] Estimated based on comparison of retention time and co-chromatography with commercial standards. GSL concentrations were calculated as µmol/g dry weight of tissue analyzed (five biological replicates per genotype).
*BnCYP79F1*_DM: *BnMYB28* double mutant (genotype $C_1C_1D_1D_1$), *BnCYP79F1*_WT: Plants with wildtype alleles for *BnCYP79F1* segregating within the same F_2 population, Express617: non-mutagenized parent
n.d: Not detectable SD: Standard deviation
